# Six anthropometric indicators and their associations with myocardial infarction and death in Swedish automotive industry male workers followed for 28 years

**DOI:** 10.1186/s12889-026-29630-0

**Published:** 2026-10-02

**Authors:** Lala Joulha Ian, Lennart Dimberg

**Affiliations:** Department of Public Health and Community Medicine, University of Gothenburg, Box 453, Gothenburg, 40530 Sweden

**Keywords:** Obesity, Body measurements, Longitudinal cohort, Myocardial infarction, Mortality, Risk factor, Sweden

## Abstract

**Background:**

Overweight, (body mass index (BMI), kg/m2) 25–30 and obesity (BMI 30+) have long been linked to an increased risk of myocardial infarction and mortality.

To better assess abdominal adiposity, several indices have been developed. However, the risk associated with each index can vary across different populations.

**Methods:**

This study aims to investigate which body measurement, if any, exhibits the strongest associations with MI, and mortality. In 1993, a cohort of 1,000 randomly selected middle-aged Swedish men participated in a nurse-led health examination. Body measurements were recorded together alongside blood pressure readings, and a fasting blood test was conducted. A follow-up study, spanning 28 years, utilized data from the national Myocardial Infarction Register (Swedeheart) and mortality data from the Swedish Cause of Death Register. The obesity indices included body mass index, waist circumference (WC), waist-to-height ratio (WHtR), waist-to-hip ratio (WHR), sagittal abdominal diameter (SAD), and sagittal abdominal diameter-to-height ratio (SADHtR). Cox proportional hazard models were employed to analyze the z-scores of the six body measurements both before and after adjusting for covariates, with outcomes assessed for first-time myocardial infarction (MI) and mortality.

**Results:**

Data were collected for 959 men. Significant correlations were found between the six body measurements, all were associated with cardiovascular disease risk factors, myocardial infarction, and mortality. However, after adjusting for the traditional risk factors, the association with cardiovascular endpoints was diminished or vanished. WHtR emerged as a more reliable indicator, with hazard ratios (HR) of 1.31 (95% CI 1.07-1.62) for myocardial infarction and 1.27 (95% CI 1.08-1.49) for all-cause mortality although the differences were marginal.

**Discussion:**

There is currently no consensus on which obesity indicator should be the preferred predictor of cardiovascular risk. In this study, the WHtR emerged as the more reliable predictor, albeit with marginal clinical significance.

**Conclusions:**

In this population, after adjustment for traditional risk factors, WHtR proved to be a more reliable predictor for MI and mortality.

**Supplementary Information:**

The online version contains supplementary material available at https://doi.org/10.1186/s12889-026-29630-0.

## The study’s important questions

### What is already known about this subject?

Different adiposity measurements have been used over time and validated as risk factors for cardiovascular disease and death in different population cohorts.

While body mass index has the longest history and the best-validated cut-offs (overweight, obesity class 1, 2, and 3), other risk factors have been argued to reflect abdominal fat better. These include waist circumference, waist-to-height ratio, sagittal abdominal diameter, sagittal diameter to height, and waist-to-hip ratio.

Later research suggests that abdominal obesity is the major risk factor.

In a recent meta-analysis of thirty-one prospective studies, no cohort was drawn from industry and results vary between different cohorts.

### What are the new findings in your manuscript?

The novelty of the study is that it addresses an important population of industrial employees and investigates six body measurements simultaneously within 28 years of follow-up. All body measurements were highly intercorrelated and correlated with known risk factors such as systolic blood pressure, smoking, and total cholesterol, which explain a substantial part of the associations.

We chose Cox proportional hazard (Cox PH) models of the z-score of the six body measures.

Waist-to-height ratio (WHtR) is a slightly superior risk index for Myocardial Infarction (MI) and death in this population. The differences using several of the other indexes were small and possibly clinically insignificant.

### How might your result change the direction of research or the focus of clinical praxis?

We found some support for a wider use of WHtR as an important cardiovascular risk index. “Keep your waist to less than half your height”, since the cut-off for WHtR is 0.5. Occupational health services would be well-positioned to advocate for lifestyle changes and identify risk factors that may be mitigated with medication.

Future studies should investigate these body measurements as risk factors in larger cohorts of industrial workers and in women.

## Background

The global obesity epidemic, often referred to as “globesity”, is a mounting concern worldwide, characterized by a body mass index (BMI) of 30 or higher. According to the Global Burden of Disease Report from 2019, nearly half of men aged 40 and above were classified as overweight or obese, with approximately 15% categorized as obese [1].

Obesity is a serious medical condition defined by the abnormal or excessive accumulation of body fat, which adversely affects health. The pathophysiology of obesity is complex, involving both subcutaneous and visceral fat.

Visceral obesity, in particular presents greater health risks than generalized obesity and is more closely linked to chronic diseases [2]. Conditions associated with visceral obesity include hypertension, diabetes, insulin resistance, and metabolic syndrome, all of which increase the risk of cardiovascular disease [3].

Cardiovascular diseases (CVDs) such as stroke, heart failure, hypertensive heart diseases, and coronary artery diseases – including angina and myocardial infarction (MI) - are the leading causes of death globally [4]. A recent 2023 study estimated that 52% of cardiovascular diseases in men are preventable or can be delayed [5].

Preventive measures against CVD include avoiding smoking, limiting alcohol consumption, maintaining a healthy diet, exercising regularly, and managing conditions such as hypertension, hyperlipidemia, and diabetes [6–8].

Despite its widespread use, BMI has notable limitations, as it does not account for critical factors like age, gender, muscle mass, and the distribution of body fat. A large meta-analysis in 2006 suggested the need for alternative methods to predict cardiovascular mortality, as BMI is only a significant predictor in cases of severe obesity [9]. Other indices, including waist circumference (WC), waist-to-height ratio (WHtR) waist-to-hip ratio (WHR), sagittal abdominal diameter (SAD), and sagittal abdominal diameter-to-height ratio (SADHtR), have been proposed as more effective measures of abdominal obesity, which is causally linked to the pathophysiology of ischemic heart disease [10, 11].

While many studies examined the relationship between body weight and various obesity indicators, with adequate follow-up duration, none of these studies focused on industrial workers in an occupational health context, and no comprehensive study has included all six body measurements in a single cohort.

The Renault-Volvo Coeur project is a prospective longitudinal investigation launched in 1992 in collaboration with the occupational health clinics of two automotive manufacturers. Its objective is to investigate national differences in cardiovascular disease particularly MI, among French and Swedish workers at Renault and Volvo [12–15].

At the outset of the Coeur project, standardized measurements for six common obesity markers were recorded. Utilizing Swedish register data from the subsequent 28 years, we can now present findings on the accumulated incidence of first MI and mortality.

This study aims to investigate which body measurement, if any, exhibits the strongest associations with MI, and mortality, employing Cox proportional hazard (Cox PH) models using the z-score of the six body measures.

## Methods

### Study population

**Fig. 1:**
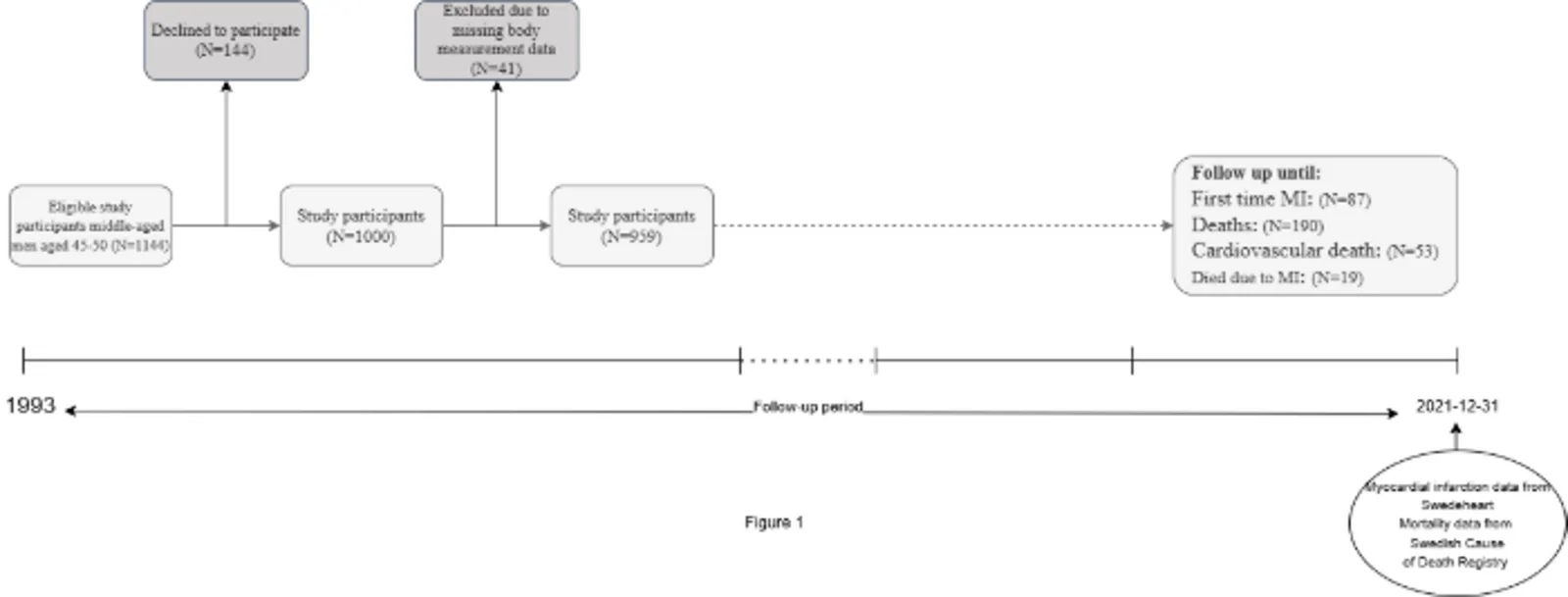
Study design

In 1993, 1144 middle-aged men (aged 45–50) from the Volvo corporation were.

randomly selected by occupational physicians using date-of-birth records and alphabetic order to participate in the study. A total of 144 employees declined to participate due to lack of interest or long-term illness. See Fig. 1 for a visual representation of the exclusion and follow-up process.

Participants underwent a nurse-led health examination that included a self-administered questionnaire, with inquiries about marital status, smoking habits, alcohol consumption, lifestyle, mental health, working conditions, stress, exercise, and other relevant factors.

The examinations were conducted at the workplace across two locations, one in Gothenburg and one in Trollhättan, approximately about 80 km from Gothenburg.

Blood pressure and heart rate were measured in a supine position as the average of three readings taken from the right arm after 10 min of resting period. Measurements were taken using a semi-automatic cuff (LIC Hygiene) in conjunction with D2 International, Hestia Pharm. Hypertension was defined as systolic blood pressure (SBP) > = 140 mm Hg, and diastolic blood pressure (DBP) > = 90 mm Hg, the use of antihypertensive treatment, or a combination of these criteria.

A venous blood sample was obtained after an overnight fast while the subject was seated, with limited use of a tourniquet. Blood samples were subsequently frozen at −20 degrees Celsius and analyzed in a single batch at Sahlgrenska University Hospital. Serum cholesterol and triglycerides were determined using classical enzymatic methods, while high-density lipoprotein cholesterol (HDL-C) was measured after precipitation of low and very low-density lipoproteins with phosphotungstic acid and magnesium chloride. Low-density lipoprotein (LDL) cholesterol was calculated using the formula: (in millimoles per liter LDL cholesterol (mmol/l)= Total cholesterol – HDL cholesterol – (Triglycerides/2.2), excluding individuals with triglycerides > 4 mmol/l.

Smoking habits were compiled by a questionnaire: The current smoker (yes/no) was defined as someone who smoked at least 1 cigarette per day for the last three months. The lifetime smoking dose was calculated in pack years, with a pack equated to 20 cigarettes; one cigar equated to two cigarettes; one cigarillo to one cigarette, and one pipe to 1.5 cigarettes.

Alcohol consumption was also recorded via the questionnaire, with total alcohol calculated as follows: one glass of beer equals 6 g, one glass of wine equals 12 g, and one glass of distilled.

spirit 13 g.

Diabetes mellitus was self-reported in the questionnaire.

The study methods have been previously detailed by Simon et al. [12].

Cardiovascular risk was estimated using the Framingham risk score, which includes age, smoking habits, SBP, diabetes mellitus, left ventricular hypertrophy, and the ratio of total cholesterol to HDL-C concentration [8].

### Body measures

BMI was calculated by dividing weight in kilograms by the square of height in meters. BMI classified according to the World Health Organization (WHO) as underweight (BMI < 18.5 kg/m²), normal weight (18.5–24.9 kg/m²), overweight (25.0–29.9 kg/m²), and obese (BMI ≥ 30 kg/m²) [16].

WC in centimeters (cm) was measured in a standing position, with the measurement tape positioned at the midpoint between the iliac crest and the lower margin of the last palpable rib [17]. Threshold values for classifying abdominal obesity were based on the National Institutes of Health guidelines [18] and classified as low risk (WC < 94 cm), high risk (WC 94–102 cm) and increased higher risk (WC > 102 cm).

The WHtR is calculated by dividing WC (cm), by height (cm). Although there is no universally accepted reference value for WHtR, an ideal ratio has been suggested to be less than or equal to 0.5 [19].

The WHR is calculated by dividing the WC (cm) by the hip circumference (cm), measured in a standing position during a soft expiration, with the measuring tape placed at the widest part of the gluteal region. The ratio determines the amount of stored fat on the waist, hips, and buttocks. WHR cut-off points according to WHO classified as low risk (WHR < 0.95), moderate risk (WHR 0.96–1.0) and high risk (WHR ≥ 1.0) [20].

The SAD is measured using a specially designed caliper determining the distance (cm) from the lower back to the highest point of the abdomen (at the belly button) while the individual lies horizontally with legs flat on the examination table. Detailed measurement procedures for SAD are described in the National Health and Nutrition Examination Survey [21].

A 2010 population-based cross-sectional study of Swedish participants aged 60 determined optimal cut-offs for SAD as 22 cm for men and 20 cm for women [22].

SADHtR serves as another screening method for assessing visceral fat and was initially proposed in an earlier phase of the Coeur project as a strong indicator of coronary risk. [23, 24]. It’s comprised of the sagittal diameter (cm) divided by the height (m). However, a limitation of SADHtR is the absence of established reference ranges and cut-off points for risk categorization.

### Outcome measures

Data on the first myocardial infarction in the Swedish cohort were obtained from the National Swedish Register of MI, Swedeheart, which is a web system that covers data across the patient pathway after an acute myocardial infarction, from hospital care to secondary prevention. [25].

MI was defined according to the International Statistical Classification of Diseases (ICD): code 410 in the ninth revision and codes I21-I23 in the tenth revision [26]. See Table 2.

Mortality data was collected from the Swedish Cause of Death Registry [27]. For cardiovascular diseases (CVD), we utilized the International Statistical Classification of Diseases: codes 390–459 in the ninth revision and codes I00-I99 in the tenth revision.

### Statistical analysis

To evaluate the relationship between anthropometric measures and the risk of myocardial infarction, as well as the risk of all-cause mortality we employed Cox proportional hazard (PH) models, both unadjusted and adjusted for relevant covariates. The model assumes that the hazard ratios between different obesity groups remain constant over time. This analysis is based on survival data, measuring the time until the event occurs for everyone in the study while accounting for individuals who may not have experienced the event during the observation period (censored data).

For each of the six anthropometric measures, the adjusted model contained the z-score of the measure along with the covariates age, SBP, smoking status, the ratio of total cholesterol to HDL-C, type of work (manual or clerical), and was stratified by marital status. The z-score provides a clear and intuitive understanding of how a group’s obesity measure compares to the average. For instance, a z-score of 1 indicates a measurement that is 1 standard deviation above the mean. Stratification was suggested by an initial analysis due to indications of non-proportionality of hazards for this covariate. We assessed the fitted models for the proportionality of hazards using scaled Schoenfeld residuals and the accompanying score test for zero slopes. Martingale residuals were used to evaluate the appropriateness of a simpler linear term for continuous covariates in the model. Hazard ratios (HRs), along with their corresponding 95% confidence intervals and p-values, are reported without adjustment for multiple testing, using a significant level of 5%.

Statistical comparisons were based on baseline variables, with detailed collection methods described by Simon et al. [12]. Baseline data for categorical (qualitative, dichotomous) variables were presented as counts and percentages, while continuous (quantitative) variables were summarized as medians with interquartile ranges (IQR) in parentheses.

To assess the association between each anthropometric measurement and cardiovascular risk factors, we calculated Spearman’s correlation coefficient. Spearman’s correlation ranges from − 1 to + 1, where values close to −1 indicate a negative monotonic relationship, zero indicates no correlation, and + 1 indicates a positive monotonic relationship. If the associated 95% confidence interval (constructed by z-transformation) included zero, the association was considered insignificant at the 5% level. For categorical variables, the distribution of each obesity variable was compared across categories using the Mann-Whitney-Wilcoxon test (for two categories) or Kruskal-Wallis (for more than two categories). The risk factor “diabetes” in the study could not be reliably analyzed due to the low number of participants (*n* = 13) at baseline.

Two-sided P values below 0.05 were deemed statistically significant. Missing values were excluded from the analyses. All analyses were performed using R (version 4.3.1, with RStudio, version 2023.03.0), utilizing the following packages rms (version 6.7-0), Hmisc (version 4.7-2) [28], survival (version 3.5–5) [29], and cmprsk (version 2.2–11) [30].

Explanatory (independent) variables included components of the Framingham risk index: age (years), SBP (mmHg), total cholesterol/HDL-C (mmol/l), smoking (No/Past/Current), and diabetes (Yes/No; excluded in the present study excluded due to low numbers [*n* = 13] at baseline). Based on information from previous studies, in this project, additional covariates included type of work (manual or clerical), and marital status [12, 15]. Manual workers are exposed to various potential hazards, including noise, chemicals, and shift work, all of which may correlate to cardiovascular risk, as explored in a Japanese study by Zaitsu et al. [31].

Outcomes include deaths from all causes, deaths due to cardiovascular causes, deaths due to MI, and the incidence of the first MI.

### Ethical approval and informed consent

This study was performed in line with the principles of the Declaration of Helsinki and included written, informed consent from all participants. The Research Ethics Committee of Gothenburg University approved the original study protocol on Feb 11, 1993 (Dnr. 23–93), and amendments on Feb 19, 2019. Pseudonymized data have been used.

Applications to use data from the National Myocardial Infarction Register, Swedeheart, and the National Cause of Death Register were also approved.

## Results

In total, 959 men had complete data on six body measurements. Among these, 60% were engaged in clerical and 38% in manual labor; 76% were married or cohabiting with a partner, and nearly one-third were current smokers.

Table 1 shows the differences between the survivors without MI, participants with MI (with or without subsequent death), and participants deceased before MI.

**Table 1:** Baseline characteristics and end-point data

Variable	Survivors without MI *N* = 697	Participants with MI (with and without subsequent death). *N* = 87	Participants deceased before MI *N* = 160
Body mass index (kg/m2), median (IQR)	25.2 (23.1–27.4)	27.1 (25.2–28.7)	25 (23.1–28.1)
Waist (cm), median (IQR)	91 (86–97)	96 (92–100)	91 (86–101)
Waist-hip ratio, median (IQR)	0.9 (0.9–1)	1 (0.9–1)	0.9 (0.9–1)
Waist-to-height ratio, median (IQR)	0.5 (0.5–0.5)	0.5 (0.5–0.6)	0.5 (0.5–0.6)
Sagittal abdominal diameter (cm), median (IQR)	20 (18–22)	21 (19.5–22)	20 (18–23)
Sagittal abdominal diameter to height ratio, median (IQR)	11.1 (10.2–12)	11.7 (10.8–12.7)	11.2 (10.2–12.7)
Age at baseline, median (IQR)	48 (46–49)	48 (47–49)	48 (47–49)
Manual workers, n (%)	248 (36%)	42 (48%)	77 (48%)
University education, n (%)	163 (23%)	25 (29%)	39 (24%)
Married or cohabitant, n (%)	561 (80%)	67 (77%)	101 (63%)
Systolic blood pressure (mmHg), median (IQR)	115 (107–123)	116 (110–130)	119 (109–132)
Diastolic blood pressure (mmHg), median (IQR)	74 (67–82)	77 (68–83)	74 (65–82)
Pulse rate (beats per minute), median (IQR)	62 (57–70)	63 (55–70)	63 (57–69)
B-Cholesterol (mmol/l), median (IQR)	5.8 (5.2–6.5)	6 (5.4–7)	5.8 (5.1–6.6)
HDL-C (mmol/l), median (IQR)	1.2 (1–1.4)	1.1 (1–1.2)	1.2 (1–1.4)
Triglycerides (mmol/l), median (IQR)	1.3 (0.9–1.8)	1.4 (1–2.1)	1.3 (1–1.9)
B-glucose (mmol/l), median (IQR)	5.4 (5–5.7)	5.6 (5–5.9)	5.4 (5.1–5.8)
Alcohol consumption gram/week, median (IQR)	39 (6–73)	40 (12–70.5)	34 (6–77)
Smoking *			
Never, n (%)	209 (30%)	9 (10%)	41 (26%)
Past, n (%)	288 (41%)	34 (39%)	49 (31%)
Current, n (%)	165 (24%)	44 (51%)	61 (38%)
Exercise *			
Less than 1 h per week, n (%)	212 (30%)	28 (32%)	52 (32%)
1–3 h of exercise/week, n (%)	331 (47%)	42 (48%)	77 (48%)
More than 3 h of exercise/week, n (%)	127 (18%)	16 (18%)	20 (13%)
Active as an athlete, n (%)	16 (2.3%)	4 (4.6%)	8 (5%)

The extent of missing values for variables denoted with * is detailed in the supplementary material (S1) where the proportion of missing values did not exceed 8%

*Abbreviations*: *N* Number of participants, *IQR* Interquartile range, *MI* Myocardial infarction, *HDL-C* High-density lipoprotein cholesterol, *B-glucose* (glucose level in blood), *B-cholesterol* (cholesterol level in blood)

An overview of the extent of missing values is presented in (Additional File 1: Table S1). Missing values vary between 0 and 8%.

The median BMI was higher in the MI group, along with WC, WHR, SAD, and SADHtR at baseline.

Compared to the survivors, systolic blood pressure was higher in the MI and death groups, remaining within normal ranges. Total cholesterol, HDL-C, triglycerides (TG), and fasting plasma glucose were similar between the groups. Current smokers were double the rate in the MI group and 50% more in the deceased group; Alcohol consumption was higher in the MI group, but lower in the deceased group; refer to Table 1 for comprehensive details.

Over the 28 years of follow-up, 87 individuals suffered at least one MI, and 190 had died, 53 of whom died due to cardiovascular causes, including 19 from MI. Table 2 shows the list of cardiovascular causes of death according to ICD-10 and ICD-9 codes.

**Table 2:** A list of cardiovascular causes of death according to ICD-10 & ICD-9 codes

Cardiovascular causes of death (ICD-10 & ICD-9)	Frequency
I110- Hypertensive heart disease with heart failure	1
I119- Hypertensive heart disease without heart failure	1
I139- Hypertensive heart and chronic kidney disease	1
I219- Acute myocardial infarction	17
I251- Atherosclerotic heart disease	8
I258- Other forms of chronic ischemic heart disease	1
I259- Chronic ischemic heart disease, unspecified	3
I269- Pulmonary embolism	2
I350- Aortic (valve) stenosis	2
I461- Sudden cardiac death	1
I482- Chronic atrial fibrillation	1
I509- Heart failure, unspecified	1
I516- Cardiovascular disease, unspecified	1
I619- Intracerebral hemorrhage, unspecified	3
I629- Intracranial hemorrhage (nontraumatic), unspecified	1
I639- Cerebral infarction, unspecified	2
I678- Other specified cerebrovascular disease	1
I709- Other and unspecified atherosclerosis	1
I710- Aortic dissection	2
I713- Abdominal aortic aneurysm, ruptured	1
410 (ICD-9)- Acute myocardial infarction	2

*Abbreviation*: *ICD* International Classification of Diseases

### Associations between the six body measures of obesity

To evaluate the relationships among the six body measurements of obesity, we employed Spearman’s correlation coefficient, as detailed in Table 3. The correlations were moderate-to-high, with the waist-to-hip ratio showing a weaker association with the other metrics.

**Table 3:** Spearman’s correlation coefficient and 95% confidence interval of six different obesity measurements

Spearman’s correlation (95%CI)	Waist circumference	Waist-to-hip ratio	Waist-to- height ratio	SAD	SADHtR
BMI	0.88 (0.86–0.89)	0.64 (0.60–0.68)	0.90 (0.88–0.91)	0.82 (0.80–0.84)	0.84 (0.82–0.86)
Waist circumference	--	0.79 (0.77–0.82)	0.93 (0.92–0.94)	0.85 (0.84–0.87)	0.81 (0.79–0.83)
Waist-to-hip ratio		--	0.83 (0.81–0.85)	0.63 (0.59–0.67)	0.66 (0.63–0.70)
Waist-to- height ratio			--	0.80 (0.78–0.82)	0.86 (0.84–0.88)
SAD				--	0.96 (0.95–0.96)
SADHtR					--

*Abbreviations*: *CI* Confidence interval, *BMI* Body mass index, *SAD* Sagittal abdominal diameter, *SADHtR* Sagittal abdominal diameter to height ratio

Table 4 shows the categorization of subjects into quartiles based on BMI, WC, WHR, WHtR, SAD, and SADHtR values. Note that the cut-off for WHtR of 0.5 is in the higher two thirds of the second quartile.

**Table 4:** Overview of the division of the subjects into quartiles

Definition of the quartile and number of individuals in the quartile	1 st quartile	2nd quartile	3rd quartile	4th quartile
BMI	< 23.2 *n* = 240	[23.2, 25.4) *n* = 241	[25.4, 27.7) *n* = 244	>=27.7 *n* = 234
Waist circumference	< 87 *n* = 254	[87, 93) *n* = 265	[93, 99) *n* = 213	>=99 *n* = 227
Waist-to-hip ratio	< 0.894 *n* = 241	[0.894, 0.933) *n* = 239	[0.933, 0.969) *n* = 241	>=0.969 *n* = 238
Waist-to-height ratio	< 0.478 *n* = 242	[0.478, 0.512) *n* = 242	[0.512, 0.549) *n* = 236	>=0.549 *n* = 239
SAD	< 19 *n* = 249	[19, 21) *n* = 312	[21, 23) *n* = 222	>=23 *n* = 176
SADHtR	< 10.3 *n* = 250	[10.29,11.2) *n* = 243	[11.18,12.2) *n* = 227	>=12.21 *n* = 239

The number of participants in the various high-risk groups varies between BMI > 30 (*n* = 95) and WHR > 0.9 (*n* = 687)

*Abbreviations*: *BMI* Body mass index, *SAD* Sagittal abdominal diameter, *SADHtR* Sagittal abdominal diameter to height ratio

The relationship between normal cut-off values of the variables and standard BMI categories is illustrated in (Additional File 1: Table S2).

### Associations between anthropometric measurements and cardiovascular risk factors

Significant associations were identified between various anthropometric measurements (excluding waist circumference), and factors such as type of work, smoking status, fasting blood glucose levels, triglycerides, HDL-C, total cholesterol, and SBP. However, p-values were not adjusted for multiple testing.

Due to missing values, the number of subjects analyzed varied slightly across different variables; this analysis is available in Tables 5 and 6.

Traditional cardiovascular risk factors, treated as continuous variables including smoking (pack-years), lipid levels, blood glucose, and SBP, exhibited significant correlations with all six indices. Similarly, categorical variables such as diabetes (yes/no), smoker (never/past/current), and manual/clerical workers status were significantly associated, although WHR was only borderline significantly associated with diabetes (*p* = 0.052), with only 13 individuals diagnosed with diabetes at study initiation.

**Table 5:** Spearman’s correlation coefficients between cardiovascular risk factors and the anthropometric measurements. Continuous variables

Spearman’s correlation (95% CI)	BMI	Waist circumference	Waist-to-hip	Waist-to-height	SAD	SADHtR
Packyears, *n* = 898	**0.15** **(0.08–0.21)**	**0.19** **(0.13–0.26)**	**0.19** **(0.12–0.25)**	**0.21** **(0.15–0.27)**	**0.17** **(0.11–0.24)**	**0.18** **(0.12–0.25)**
Alcohol consumption, *n* = 933	0.04 (−0.03–0.10)	0.02 (−0.04–0.09)	−0.01 (−0.07–0.06)	0.02 (−0.04–0.09)	0.01 (−0.05–0.08)	0.01 (−0.05–0.08)
B-glucose, *n* = 919	**0.28** **(0.21–0.33)**	**0.26** **(0.20–0.32)**	**0.17** **(0.11–0.24)**	**0.24** **(0.18–0.30)**	**0.24** **(0.18–0.30)**	**0.22** **(0.16–0.28)**
Triglycerides, *n* = 938	**0.40** **(0.34–0.45)**	**0.42** **(0.36–0.47)**	**0.35** **(0.29–0.40)**	**0.41** **(0.36–0.46)**	**0.44** **(0.38–0.49)**	**0.44** **(0.38–0.49)**
HDL-C, *n* = 892	**−0.36** **(−0.41–0.30)**	**−0.34** **(−0.40–0.28)**	**−0.25** **(−0.31 −0.18)**	**−0.35** **(−0.4–0.29)**	**−0.33** **(−0.39–0.27)**	**−0.34** **(−0.40–0.28)**
B-cholesterol, *n* = 940	**0.19** **(0.13–0.25)**	**0.21** **(0.15–0.27)**	**0.20** **(0.13–0.26)**	**0.23** **(0.17–0.29)**	**0.22** **(0.15–0.28)**	**0.23** **(0.17–0.29)**
Pulse, *n* = 930	−0.01 (−0.07–0.06)	0.02 (−0.04–0.09)	0.01 (−0.05–0.07)	0.00 (−0.06–0.07)	0.03 (−0.03–0.10)	0.02 (−0.04–0.09)
SBP, *n* = 959	**0.22** **(0.16–0.28)**	**0.22** **(0.16–0.28)**	**0.15** **(0.08–0.21)**	**0.20** **(0.14–0.26)**	**0.29** **(0.23–0.34)**	**0.27** **(0.21–0.33)**
DBP, *n* = 936	0.01 (−0.06–0.07)	0.02 (−0.04–0.09)	0.01 (−0.05–0.07)	0.00 (−0.06–0.07)	0.03 (−0.03–0.09)	0.02 (−0.04–0.09)

The 95% confidence intervals are provided and statistically significant results are highlighted in bold

*Abbreviations*: *CI* Confidence interval, *N* Number, *BMI* Body mass index, *SAD* Sagittal abdominal diameter, *SADHtR* Sagittal abdominal diameter to height ratio, *B-glucose* (glucose level in blood), *HDL-C* High-density lipoprotein cholesterol, *B-Cholesterol* (cholesterol level in blood), *SBP* Systolic blood pressure, *DBP* Diastolic blood pressure

**Table 6:** Association of the anthropometric measurements with different cardiovascular risk factors, and categorical variables

Median [IQR], *p*-value	BMI	Waist circumference	Waist-to-hip	Waist-to- height	SAD	SADHtR
Diabetes No, *n* = 947 Yes, *n* = 12	***p*** ** = 0.03**	***p*** ** = 0.03**	*p* = 0.052	***p*** ** = 0.03**	***p*** ** = 0.03**	***p*** ** = 0.03**
	25.3 [23.2–27.6]	92 [86–98]	0.93 [0.89–0.97]	0.51 [0.48–0.55]	20 [18–22]	11.2 [10.3–12.2]
	28.2 [24.9–30.0]	97 [92–107]	0.95 [0.92–1.02]	0.54 [0.51–0.60]	21.5 [20 − 23.2]	12.2 [11.0–13.3]
Smoker Never, *n* = 259 Past, *n* = 371 Current, *n* = 270	***p*** ** = 0.005**	***p*** ** = 0.001**	***p*** ** = 0.02**	***p*** ** = 0.002**	***p*** ** = 0.0003**	***p*** ** = 0.001**
	24.8 [23.1–26.7]	90 [85–96]	0.93 [0.89–0.96]	0.51 [0.47–0.53]	20 [18–22]	10.9 [9.9–12.0]
	25.9 [23.6–28.0]	93 [87–99]	0.94 [0.90–0.97]	0.52 [0.48–0.55]	20 [19–22]	11.4 [10.5–12.3]
	25.2 [23.1–27.5]	92 [86–98]	0.94 [0.90–0.97]	0.51 [0.48–0.55]	20 [19–22]	11.1 [10.4–12.1]
Married No, *n* = 229 Yes, *n* = 729	*p* = 0.10	*p* = 0.18	*p* = 0.6	*p* = 0.7	*p* = 0.2	*p* = 0.5
	25.1[22.9–27.5]	91 [86–98]	0.93 [0.89–0.97]	0.51 [0.47–0.55]	20 [18–22]	11.0 [10.1–12.2]
	25.4[23.4–27.8]	92 [86–98]	0.93 [0.90–0.97]	0.51 [0.48–0.55]	20 [19–22]	11.2 [10.3–12.2]
University education No, *n* = 707 Yes, *n* = 227	*p* = 0.8	*p* = 0.7	*p* = 0.8	*p* = 0.7	*p* = 1.0	*p* = 0.9
	25.4 [23.3–27.7]	92 [86–98]	0.93 [0.89–0.97]	0.51 [0.48–0.55]	20 [18–22]	11.2 [10.3–12.2]
	25.4 [23.1–27.5]	92 [87–98]	0.93 [0.89–0.96]	0.51 [0.48–0.55]	20 [18.5–22]	11.1 [10.4–12.1]
Type of work clerical, *n* = 577 manual, *n* = 367	***p*** ** = 0.04**	*p* = 0.1	***p*** ** = 0.01**	***p*** ** = 0.001**	***p*** ** = 0.03**	***p*** ** = 0.001**
	25.1 [23.1–27.5]	91 [86–97]	0.93 [0.89–0.96]	0.51 [0.48–0.54]	20 [18–22]	11.0 [10.1–12.1]
	25.7 [23.3–27.8]	92 [86–99]	0.94 [0.90–0.97]	0.52 [0.48–0.55]	20 [19–22]	11.3 [10.5–12.5]
Exercise No, *n* = 292 1–3 h/week, *n* = 450 > 3 h/week, *n* = 163 Athlete, *n* = 28	*p* = 0.2	*p* = 0.7	*p* = 0.8	*p* = 0.5	*p* = 0.4	*p* = 0.5
	25.2 [23.4–27.1]	91.5 [86–98]	0.93 [0.89–0.97]	0.51 [0.48–0.55]	20 [18–22]	11.1 [10.2–12.1]
	25.4 [23.1–27.8]	92 [86–98]	0.93[0.89–0.97]	0.51 [0.48–0.55]	20 [19–22]	11.2 [10.4–12.3]
	25.1 [23.5–27.5]	92 [87–98]	0.93 [0.90–0.97]	0.51 [0.49–0.55]	20 [19–22]	11.2 [10.4–12.0]
	27.3 [24.2–29.2]	93 [87.8–102]	0.94 [0.91–0.97]	0.53 [0.50–0.56]	20 [18.8–22]	11.3 [10.6–12.5]

The median and interquartile range of the anthropometric measurements within each category of the risk factor is shown. Statistically significant results are highlighted in bold

*Abbreviations*: *N* Number, *BMI* Body mass index, *SAD* Sagittal abdominal diameter, *SADHtR* Sagittal abdominal diameter to height ratio, *IQR* Interquartile range, *p-value* Probability value

### Cox proportional hazard ratio models containing z-score and myocardial infarction

Among 959 participants, there were 87 instances of MIs and 19 deaths due to MI.

Table 7 shows unadjusted and adjusted hazard ratios (HR) with 95% confidence intervals for a change by one standard deviation in the anthropometric indicators. The adjustment was done for age, systolic blood pressure, smoking, the ratio of total cholesterol to HDL-C, type of work (manual/clerical), and marital status.

**Table 7:** Hazard ratio for myocardial infarction. The table shows unadjusted and adjusted hazard ratios (HR) with 95% confidence intervals for a change by one standard deviation in the anthropometric indicators. The adjustment was done for age, systolic blood pressure, smoking, ratio of total cholesterol to HDL-C, type of work (blue/white collar), and marital status

Anthropometric measure (z-score)			Unadjusted HR (95%CI)	*p*-value	Adjusted HR (95%CI)	*p*-value
BMI			1.39 (1.18–1.63)	< 0.0001	1.22 (0.99–1.52)	0.0684
Waist circumference			1.46 (1.23–1.72)	< 0.0001	1.28 (1.03–1.6)	0.0269
Waist-to-hip ratio			1.39 (1.19–1.63)	< 0.0001	1.28 (1.04–1.57)	0.0199
Waist-to-height ratio			1.50 (1.29–1.74)	< 0.0001	1.31 (1.07–1.62)	0.0107
SAD			1.31 (1.12–1.54)	0.0008	1.13 (0.9–1.43)	0.2919
SADHtR			1.36 (1.16–1.58)	0.0001	1.16 (0.93–1.45)	0.1895
Detailed results: Note that since the adjustment for marital status was done by stratification, no HR for this covariate is available. The section below shows detailed results of HRs by anthropometric measures per co-variate.						

Adjusted Cox PH models for MI	BMI	Waist circumference	Waist-to-hip	Waist-to-height	SAD	SADHtR
Zscore	1.22(0.99–1.51)	1.28 (1.03–1.6)	1.28 (1.04–1.57)	1.31 (1.07–1.62)	1.13 (0.9–1.43)	1.16 (0.93–1.45)
Age	1.16(1.00–1.35)	1.15 (0.99–1.35)	1.15 (0.99–1.34)	1.15 (0.99–1.34)	1.16 (0.99–1.35)	1.16 (0.99–1.35)
Former smoker	2.45(1.13–5.35)	2.4 (1.1–5.22)	2.48 (1.13–5.44)	2.4 (1.1–5.22)	2.45 (1.12–5.36)	2.44 (1.12–5.34)
Current smoker	5.45 (2.48–11.98)	5.35 (2.44–11.73)	5.4 (2.45–11.91)	5.34 (2.44–11.72)	5.4 (2.46–11.85)	5.4 (2.46–11.85)
Cholesterol ratio	1.22 (1.08–1.37)	1.22 (1.08–1.36)	1.23 (1.1–1.38)	1.21 (1.07–1.36)	1.24 (1.1–1.39)	1.23 (1.09–1.38)
SBP	1.02 (1.00–1.03)	1.02 (1.00–1.03)	1.02 (1.00–1.03)	1.01 (1.00–1.03)	1.02 (1.00–1.03)	1.02 (1.00–1.03)
Manual work	1.4 (0.88–2.22)	1.41 (0.89–2.25)	1.37 (0.86–2.17)	1.37 (0.86–2.17)	1.41 (0.89–2.23)	1.38 (0.87–2.2)

*Abbreviations*: *BMI* Body mass index, *SAD* Sagittal abdominal diameter, *SADHtR* Sagittal abdominal diameter to height ratio, *Total chol/HDL-C*,* HR* Hazard ratio, *CI* Confidence intervals, *p-value*, Probability value, *MI* Myocardial infarction, *SBP* Systolic blood pressure, *Cox PH* Cox proportional hazard

All anthropometric measures showed statistically significant associations with the hazard of myocardial infarction in the unadjusted analysis, with comparable hazard ratios. After adjustment, only WHtR, WHR, and WC in that ranking order, remained statistically significant. The estimated HR suggested about 30% higher hazard for myocardial infarction per one standard deviation increase in these measurements. SAD, SADHtR, and BMI were not significantly associated with the hazard ratio.

Table 8 shows the comparison of distribution (median, interquartile range) of anthropometric measures between individuals who died from cardiovascular causes including myocardial infarction, and those who died from other causes. Significant associations with mortality due to cardiovascular causes were observed for all body measures except the waist-to-hip ratio (see Table 8).

**Table 8:** Comparison of the distribution of anthropometric measures between individuals who died from cardiovascular causes including myocardial infarction and those who died from other causes. Unadjusted data

Median (interquartile range)	Death due to all cardiovascular causes *n* = 53	Death due to other causes *n* = 137	*p*-value
BMI	27.8 (24.7–29.7)	25.1 (23.1–27.2)	0.001
Waist circumference	98 (91–103)	91 (86–99)	0.004
Waist-to-hip ratio	0.95 (0.93–0.99)	0.94 (0.90–0.98)	0.2
Waist-to-height ratio	0.55 (0.51–0.58)	0.51 (0.48–0.55)	0.002
SAD	22 (20–23)	20 (18–22)	0.01
SADHtR	12.1 (10.9–13.1)	11.1 (10.3–12.3)	0.01

*Abbreviations*: *BMI* Body mass index, *SAD* Sagittal abdominal diameter, *SADHtR* Sagittal abdominal diameter to height ratio, *p-value* Probability value

### Results of the Cox PH models containing z-score for all-cause mortality

Of 959 participants, 190 died within this period. Table 9 shows unadjusted and adjusted hazard ratios (HR) with 95% confidence intervals for changes by one standard deviation in the anthropometric indicators. Adjustments were made for age, systolic blood pressure, smoking status, ratio of total cholesterol to HDL-C, type of work (manual or clerical), and marital status. For all-cause mortality, all anthropometric measures demonstrated significant associations with HRs of mortality with values being quite similar across the measures. The WHtR showed a slightly higher HR. After adjusting for confounding factors, the strongest HRs were observed for WHtR and WC in that order both with the same HR = 1.27, but WHtR accompanied by narrower confidence intervals (CI).

**Table 9:** Hazard ratios for all-cause mortality. The table shows unadjusted and adjusted hazard ratios (HR) with 95% confidence intervals for a change by one standard deviation in the anthropometric indicators. The adjustment was done for age, systolic blood pressure, smoking, ratio of total cholesterol to HDL-C, type of work (blue/white collar), and marital status

Anthropometric measure (z-score)			Unadjusted HR (95%CI)	*p*-value	Adjusted HR (95%CI)	*p*-value
BMI			1.19(1.02–1.39)	0.0246	1.22 (1.03–1.43)	0.0183
Waist circumference			1.26(1.08–1.47)	0.0032	1.27 (1.08–1.5)	0.0044
Waist-to-hip ratio			1.27(1.10–1.46)	0.0009	1.19 (1.03–1.38)	0.0177
Waist-to-height ratio			1.27 (1.10–1.48)	0.0011	1.27 (1.08–1.49)	0.0039
SAD			1.22 (1.06–1.42)	0.0073	1.22 (1.04–1.44)	0.0179
SADHtR			1.25(1.08–1.44)	0.0035	1.23 (1.04–1.45)	0.0135
Detailed results: Note that since the adjustment for marital status was done by stratification, no HR for this covariate is available. The section below shows detailed results of HRs by anthropometric measures per co-variate.						

Adjusted Cox PH models for all-cause mortality	BMI	waist	Waist-to-hip	Waist-to-height	SAD	SADHtR
Zscore	1.22 (1.03–1.43)	1.27 (1.08–1.5)	1.19 (1.03–1.38)	1.27 (1.08–1.49)	1.22 (1.04–1.44)	1.23 (1.04–1.45)
Age	1.19 (1.07–1.32)	1.18 (1.06–1.31)	1.17 (1.06–1.31)	1.18 (1.06–1.31)	1.18 (1.06–1.31	1.18 (1.06–1.31)
Former smoker	0.78 (0.52–1.18)	0.77 (0.51–1.15)	0.79 (0.53–1.19)	0.77 (0.51–1.16)	0.77 (0.51–1.16)	0.77 (0.51–1.16)
Current smoker	2.02 (1.37–2.99)	1.97 (1.33–2.91)	1.95 (1.32–2.89)	1.97 (1.33–2.91)	1.99 (1.35–2.95)	1.99 (1.35–2.94)
Cholesterol ratio	0.91 (0.81–1.02)	0.9 (0.81–1.01)	0.92 (0.83–1.03)	0.9 (0.8–1.01)	0.91 (0.82–1.02)	0.91 (0.81–1.01)
SBP	1.02 (1.01–1.03)	1.02 (1.01–1.03)	1.02 (1.01–1.03)	1.02 (1.01–1.03	1.02 (1.01–1.03)	1.02 (1.01–1.03)
Manuel work	1.48 (1.08–2.02)	1.48 (1.09–2.03)	1.45 (1.06–1.98)	1.45 (1.06–1.98)	1.48 (1.08–2.02)	1.46 (1.07–1.99)

*Abbreviations*; *BMI* Body mass index, *SAD* Sagittal abdominal diameter, *SADHtR* Sagittal abdominal diameter to height ratio; Total chol/HDL-C, *HR* Hazard ratio, *CI* Confidence intervals, *p-value* Probability value, *MI* Myocardial infarction, *SBP* Systolic blood pressure, *Cox PH* Cox proportional hazard

## Discussion

The study aims to assess which body measurement, if any, exhibits the strongest associations with MI, and mortality. In the Cox PH analyses, the strongest hazard for MI based on z-scores was observed for WHtR followed closely by WC and WHR.

For all-cause mortality, all anthropometric measures were significantly associated with the hazard, with WHtR tying for the highest HR alongside WC, thereafter BMI and WHR followed.

Notably, WHtR emerged as the most pertinent index for both MI and all-cause mortality, though confidence intervals among the indices largely overlapped.

BMI was significantly associated with MI both before and after adjustment, but not with all-cause mortality post-adjustment.

Recently, three meta-analyses examining cardiovascular risk indicators (not specifically focusing on MI) produced conflicting results. Cao et al. identified WHR as the superior indicator for cardiovascular risk, while Xue et al. suggested WC, WHR, and WHtR had the best predictive capabilities. De Koning et al. attributed equal importance to WHR and WC [32–34]. The study of Zhang pointed out that WHtR had the highest association with coronary heart diseases in 4,244 rural Chinese men with hypertension followed for nearly five years [35].

Another cross-sectional study of 26,701 middle-aged Chinese men, pointed out that WHtR had a slightly stronger association with cardiovascular health than WC [36].

Other studies also demonstrated that WHtR was associated with an increased hazard of CVD and all-cause mortality [37], The study of Ashwell also supports the simple message “Keep your waist to less than half your height”. The study also indicates that the use of WHtR may help to live longer [38].

### Intercorrelations among all body measurements and covariates

While all body measurements exhibited significant intercorrelations, WHR showed a somewhat weaker correlation. This finding is consistent with a study of 4,504 participants from 29 countries [39].

A significant correlation was evident among all six indicators and covariates, except for total alcohol consumption, pulse, and DBP. Previous studies have highlighted similar associations with risk factors and debated which body measures are strongest associated [40, 41].

Visceral obesity is widely recognized as a critical cardiovascular risk factor, especially in cases of extreme obesity [42].

A systematic review of the impacts of both waist and hip circumference underscored the differences in fat distribution; waist circumference primarily reflects visceral and subcutaneous fat around the abdomen, while hip circumference accounts for both adipose tissue and lower body mass [43].

Regarding cardiovascular risk linked to inflammation and the secretion of inflammatory markers, as well as the pathophysiology of ischemic heart disease, the strong associations observed in our study suggest that abdominal fat may significantly impact blood pressure, blood lipids, and smoking behavior [3, 11].

### Major risk predictor models

In a cardiovascular risk evaluation intended for primary care, the authors of the Framingham.

heart study (2008) followed 8491 participants and did not include BMI in their risk prediction.

model which included gender, age, blood pressure, smoking, and lipid levels. They noted,

however, that a simplified office-based CVD risk prediction model that included body mass index (instead of total and HDL cholesterol) performed reasonably well [44].

In agreement with these findings, WHO (2025) recommends utilizing BMI in scenarios where lipid variables are not available [45].

The SCORE project (2003), estimates a 10-year risk of fatal cardiovascular events in.

Europe did not prioritize any obesity indicator in its algorithm [46].

The data in our study, however, indicate that the ratio of waist circumference to height is the strongest predictor of MI and mortality in this population, even after adjustment for covariates.

Notably, basketball star Michael Jordan’s BMI fluctuated between 27 and 29, yet his waist size was merely 76 cm (low risk). For him, muscle mass, rather than fat mass, contributed to the elevated BMI, suggesting that WC or WHtR would have been a more relevant measure than BMI [47].

One could argue that certain cardiovascular risk factors may be a consequence of obesity and, therefore, should not be adjusted for. The univariable results are provided for the reader’s review.

### Study strength

The original cohort was randomly selected from a well-characterized population of male workers followed over 28 years. The participation rate was exceptionally high, with very few individuals refusing to take part, indicating a strong level of cooperation. Nurses and health professionals were specially trained and well-defined methodologies were employed to ensure data accuracy [12].

Endpoints such as death and myocardial infarction were naturally captured through established national registers, which are considered reliable sources [25, 27]. The internal validity of the current project has been meticulously maintained throughout the study, reinforcing the robustness of the findings.

### Study weaknesses and limitations

The cohort was restricted to middle-aged men from a specific occupational sector (automotive) in Sweden, limiting the external validity of the findings to other populations, including women and individuals in different contexts. This constraint also narrows the scope of the events assessed. Additionally, some data were incomplete due to manual entry errors or missing laboratory results.

The Swedeheart registry collects MI data primarily from hospitals and intensive care units. During the early years of our study when the register was first implemented, there may have been underreporting, Cases of MI that did not result in hospitalization and sudden deaths due to MI being not captured [25]. In Sweden, most deaths do not undergo autopsy. For instance, ICD I25 (chronic ischemic heart disease) in the National death registry may not always reflect the direct cause of death. Furthermore, the limited number of participants and endpoints may have hindered our ability to detect significant risk differences between various body measurements. As in all studies, there is a possibility of spurious findings.

### Confounding factors and biases

Longitudinal studies are susceptible to confounding factors and biases, particularly as participants’ lifestyles and health statuses can change over time. For instance, over 28 years, individuals may gain or lose weight or alter their smoking habits, which can affect the prognosis of various endpoints., or quit smoking, which may change the prognosis of some endpoints.

Self-reported data in questionnaires can introduce bias, as individuals may underestimate their smoking or alcohol consumption, leading to systematic errors.

Additionally, body measurements may be subject to inaccuracies due to measurement techniques, although extensive training for health professionals was mandated before the study commenced.

## Conclusion

Our analysis revealed significant correlations among the six different body measurements, all were associated with CVD risk factors such as SBP, diabetes, and dyslipidemia, as well as smoking. All anthropometric measures showed statistically significant associations with the hazard of myocardial infarction in the unadjusted analysis, with comparable hazard ratios. After adjustment for covariates, WHtR, WHR, and WC, in that ranking order, remained statistically significant, with the hazard ratios indicating approximately a 30% higher risk for MI per one standard deviation increase associated with the hazard.

Similarly, the Cox PH models containing z-scores for all causes of death indicated that all anthropometric measures were significantly associated with the hazard of all-cause mortality, with estimated hazard ratios closely aligned; WHtR appears to be a slightly superior risk index for both MI and death in this population, although the differences among other indices were minimal and possibly clinically insignificant.

Future studies should investigate these body measurements as risk factors in larger cohorts of industrial workers and women. Additionally, the effectiveness of occupational health services in mitigating risk behaviors warrants further evaluation.

## Supplementary Information

Supplementary Material 1.

## Data Availability

The data for this study is available upon request from the corresponding author. The request should state the title and aim of the research for which the data are requested.

## Abbreviations

BMI: Body mass index
CI: Confidence interval
Cox PH: Cox proportional hazard
CVDs: Cardiovascular diseases
DBP: Diastolic blood pressure
HDL-C: High-density lipoprotein cholesterol
HR: Hazard ratio
ICD: International classification of disease
IQR: Interquartile ranges
LDL: Low-density lipoprotein
MI: Myocardial infarction
OR: Odds ratio
P-Value: Probability value
SAD: Sagittal abdominal diameter
SADHtR: Sagittal abdominal diameter to height ratio
SBP: Systolic blood pressure
TG: Triglycerides
WC: Waist circumference
WHO: World Health Organization
WHR: Waist-to-hip ratio
WHtR: Waist-to-Height Ratio
